# Digital strategies to promote crowdsourcing open calls for co-creating HIV interventions: a youth community-based participatory approach

**DOI:** 10.3389/fdgth.2025.1608366

**Published:** 2025-07-15

**Authors:** Olaoluwaposi Ogunlana, Peter Kalulu, Ucheoma Nwaozuru, Olufunto A. Olusanya, Oluwayemisi T. Olaoluwa, Temitope Ojo, Titilola Gbaja-Biamila, Chiyere Arinze, Lateef Akeem, Lauren Fidelak, Chisom Obi-Jeff, Oliver C. Ezechi, Joseph D. Tucker, Juliet Iwelunmor

**Affiliations:** ^1^ College of Medicine, University of Ibadan, Ibadan, Nigeria; ^2^ Division of Infectious Diseases, John T. Milliken Department of Medicine, School of Medicine, Washington University in St. Louis, St. Louis, MO, United States; ^3^ Department of Implementation Science, School of Medicine, Wake Forest University, Winston-Salem. NC, United States; ^4^ Department of Psychology, Faculty of the Social Sciences, University of Ibadan, Ibadan, Nigeria; ^5^ Clinical Sciences Department, Nigerian Institute of Medical Research (NIMR), Lagos, Nigeria; ^6^ Department of Medicine, Institute for Global Health and Infectious Diseases, University of North Carolina at Chapel Hill, Chapel Hill, NC, United States; ^7^ Brooks Insights, Abuja, Federal Capital Territory, Nigeria; ^8^ Department of Clinical Research, Faculty of Infectious and Tropical Diseases, London School of Hygiene and Tropical Medicine, London, United Kingdom

**Keywords:** youth participatory action research, crowdsourcing, digital engagement, implementation strategies, adolescents and young adult (AYA), HIV interventions

## Abstract

**Introduction:**

The HIV epidemic disproportionately affects adolescents and young adults (AYA), yet their engagement in HIV programming remains limited. Digital strategies, such as social media campaigns, engage a diverse range of AYA to co-create HIV interventions, but their effectiveness is less known. This study examines the digital strategies employed to engage AYA (ages 14–24) in the participatory design of HIV research and interventions in Nigeria.

**Methods:**

We employed youth participatory action research (PAR), specifically using a crowdsourcing open call strategy to generate innovative ideas from AYA on how community-based organizations can sustain youth-friendly HIV services for at-risk youth. Crowdsourcing involves a group of people solving a problem and then sharing selected solutions with the public. The open call was held between February and March 2024 as part of the Sustaining Innovative Tools to Expand Youth-Friendly HIV Self-Testing (S-ITEST) study. The open call was promoted on social media platforms and through peer youth ambassadors. Submissions were received via various channels, including Google Forms, WhatsApp, and in-person submissions. Social media engagement metrics and open call submission data were collected and analyzed descriptively using R version 4.4.2.

**Results and discussion:**

Using social media metrics, the first Instagram post reached 310 people (75% of followers) and generated 43 engagements. The second post reached 272 people (79% of followers) with 29 interactions. The first and second Facebook posts reached 153 and 58 people, respectively, with each post receiving five interactions. We received 123 submissions, with 104 submitted online. Youth in all six geopolitical zones submitted ideas (40 males and 64 females). The mean age of participants was 21.9 (SD =5.35), and most (81%) learned about the open call through digital channels, with WhatsApp (*n* = 20) being the most common channel. Older youth were more likely to hear about the open call digitally (Mean = 22.5; SD = 5.12) compared to younger participants (Mean = 19.8; SD = 5.87). Building digital communities and opportunities could sustain youth involvement in HIV research. Our findings suggest that digital strategies can complement and optimize in-person engagement to effectively leverage AYA's creativity in co-creating HIV interventions in low-resource settings.

## 1 Introduction

According to the Joint United Nations Programme on HIV/AIDS (UNAIDS), over 40 million people have died from AIDS-related illnesses since the onset of the pandemic (1). Although new HIV infections have declined by over 60% since the peak of the epidemic in 1995 (1), recent figures show the global community is far from achieving the target to end the epidemic by 2030 (2, 3). Additionally, hundreds of thousands still acquire the virus yearly, with adolescents and young adults (AYA) highly vulnerable (1, 3). In 2023, about 360,000 AYA aged 15 and 24 acquired HIV (3). In Nigeria alone, over 150,000 adolescents and young adults are living with HIV, with low rates of HIV testing and linkage to care among this age group (4). UNAIDS emphasizes engaging AYA as key stakeholders in the HIV response, not just as beneficiaries, describing it as a key area to guide HIV interventions (2, 5). This is partly because AYA are characterized by unique behavioral and psychological changes that may increase their risk of acquiring HIV (6). For instance, they may engage in high-risk activities such as having multiple sexual partners, practicing unprotected sex, and using substances (7). Many of them also lack access to HIV testing and prevention services, primarily due to sociocultural factors that limit their autonomy in making decisions about sexual health (8).

AYA have immense potential to drive change, especially in regions most affected by HIV. However, they face several barriers that prevent their meaningful engagement in the HIV response, such as stigma and lack of access to research opportunities despite having the right to participate in decision-making regarding their health (9–12). Moreover, AYA under 25 make up about 60% of Africa's population, where 26 million (64%) out of the 40 million people living with HIV globally reside (9, 13). Involving AYA in designing HIV research and interventions ensures a sustainable and effective response (5, 13). Youth engagement in HIV research and intervention is not new in Africa. Some sexual health initiatives have collaborated with AYA to design adolescent and youth-friendly HIV services (14, 15). Across the world, researchers have incorporated digital strategies through social media to tackle HIV by promoting awareness, HIV testing, digital peer support, and research participation (15–19). In Nigeria, projects like the ICARE study utilized social media as an avenue to connect young men who have sex with men with HIV testing services through peer navigators (20).

Widespread internet adoption could provide new opportunities to engage AYA in designing HIV research and interventions. In low-income regions like Africa, AYA (ages 14–24) are increasingly becoming ‘digital natives,’ being twice as likely to use the Internet as the general population and their peers in high-income regions, and having grown up with social media and instant messaging apps like Facebook Messenger and WhatsApp (21). A study among adolescents in Ibadan, Nigeria, showed that many students began using the internet at 12 years and younger (22). Social media platforms can serve as strategies to engage AYA in designing HIV interventions and research, creating youth-friendly and sustainable services (23). Social media platforms like Facebook, Twitter, and Instagram allow researchers to reach diverse and hard-to-reach populations, including AYA, marginalized communities, and key populations at risk of HIV (24). Although print media can also be used as a tool for community engagement, it is slowly becoming inaccessible in our increasingly digital society, and it typically does not allow direct engagement with an audience (25). In our study, social media is used as a tool to engage AYA in designing HIV research or interventions.

Combining in-person engagement with digital strategies ensures inclusivity, particularly for youth at high risk of acquiring HIV, and offers a convenient way to reach a broad audience (26). Moreover, actively engaging AYA provides AYA with valuable knowledge and skills about HIV prevention and treatment to prepare them as future health leaders (2, 23). In this paper, we assessed digital strategies for engaging AYA in designing HIV interventions to sustain youth-friendly HIV preventive services for at-risk AYA.

## 2 Methods

This study assesses digital strategies for engaging AYA in the participatory design of youth-friendly HIV prevention interventions and research.

### 2.1 Study design

**Figure 1:**
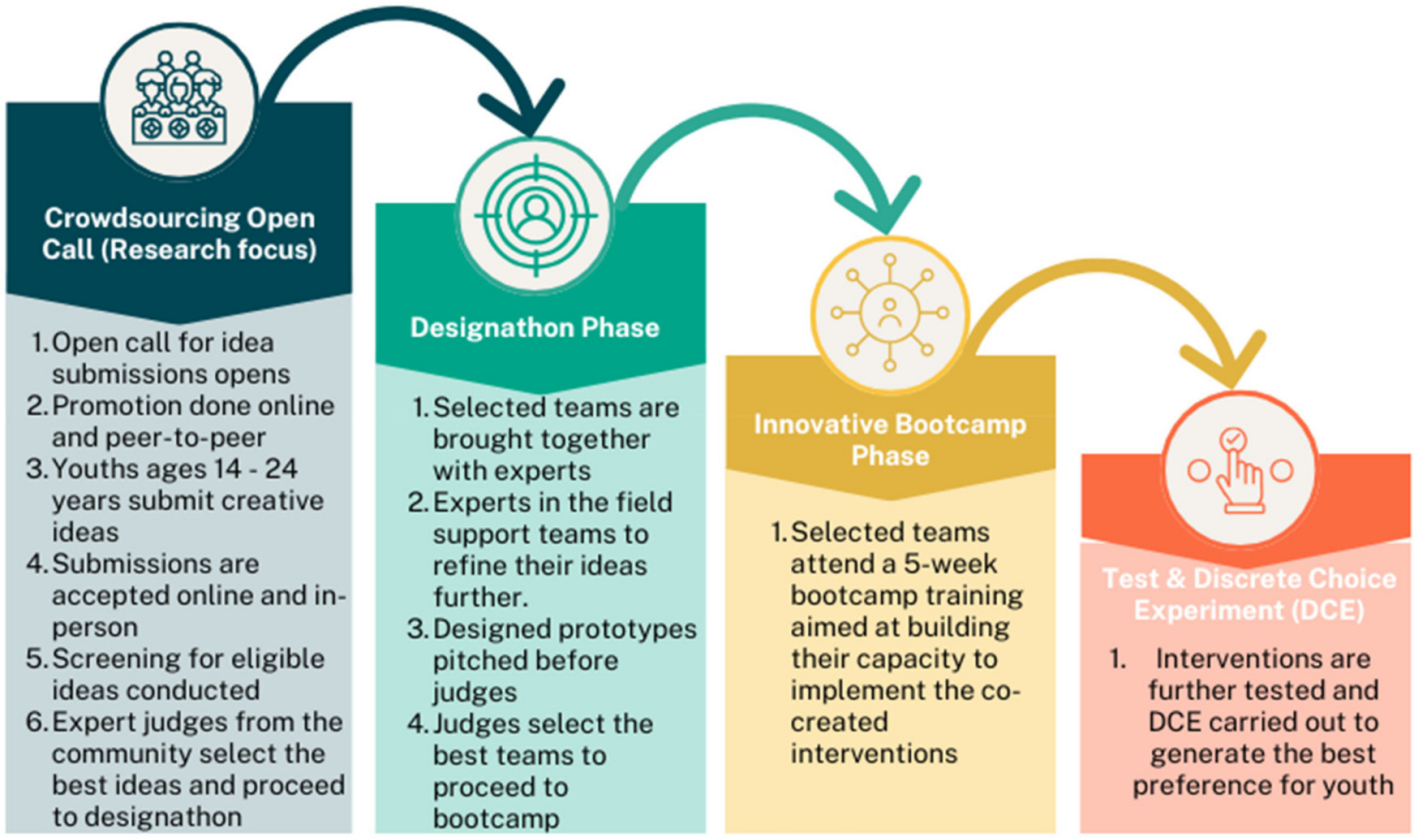
Overview of the youth participatory process.

We employed a convergent mixed-methods approach, which simultaneously collected both quantitative and qualitative data. This study is part of the S-ITEST study, which utilizes participatory implementation science approaches, including crowdsourcing, designathons, and participatory learning communities. The crowdsourcing open call, which is the focus of this research, represented the study's first phase. The second and third phases involved the designathon and the bootcamp (see Figure 1), which are detailed in other published manuscripts.

### 2.2 Crowdsourcing open call, participants’ identification, and recruitment strategies

An open call with the prompt, “*How might we use assets within youth groups and community-based organizations to sustain youth-friendly HIV self-testing (HIVST) and preventive services?”* was hosted for two months from February to March 2024 to ensure that a broad audience of potential participants was reached. The goal was for young Nigerians (ages 14–24) to submit their ideas on how community-based organizations can sustain youth-friendly HIV prevention services for at-risk youths. An open call is an avenue for experts to source ideas on solutions to specific problems from large groups using a crowdsourcing model (27, 28). The open call was promoted online through the 4 Youth by Youth (4YBY) social media channels, including Instagram and Facebook, which have largely youth followers, as well as WhatsApp, X (formerly known as Twitter), and other channels. This promotion was also facilitated through youth ambassadors sharing information with their peers and distributing flyers in the community. These youth ambassadors, members of the Youth Advisory Board, hold significant influence among their peers. The Youth Advisory Board (YAB) plays a vital role in the participatory activities of the study, consisting of six adolescents and young adults aged 14–24 who represent the six geopolitical zones in Nigeria. Members, including current youth ambassadors and other applicants selected through a competitive process, actively engage in promoting the open call both online and in person, evaluate submissions, judge entries, and share youth perspectives during steering committee discussions. Specifically, the YAB has contributed to various aspects of the open call, including its design, the development of outreach strategies, and promotional efforts, particularly through digital channels such as social media (WhatsApp, Instagram, LinkedIn, and Facebook), as well as in-person events at churches and universities. They collaborate closely with the Social Media Manager to create relevant content for the open call, ensuring that youth voices are integrated throughout all stages of the process.

### 2.3 Submissions, screening, and participant selection

Submissions were received through various channels; for example, youth were encouraged to submit their ideas through online Google form submissions, WhatsApp, and in-person submissions. Using pre-defined criteria, such as ideas being well-described, desirability to the youth, innovativeness, and feasibility, an expert panel of judges screened eligible submissions anonymously. Ten exceptional teams across Nigeria were selected by the panel of judges and invited to proceed to the designathon phase in Lagos.

### 2.4 Digital strategies

Table 1 presents digital strategies employed to engage the youth. Instagram and Facebook were the primary digital platforms utilized to connect with youth. The project team published the open call on the 4YBY Instagram and Facebook pages. All posts were published on both platforms on the same day, February 5th, 2024, with varying times for each platform's first and second posts. The 4YBY Instagram page, which had approximately 4,443 followers, shared its first post at 18:23 and its second at 18:31 West African Time. Additionally, for the 4YBY Facebook page, which had around 1,000 followers, the team published the first post at 18:24 and the second at 18:31.

**Table 1:** Digital strategies used to reach the youth during the open call.

Digital platform	Post date and time		Followers
	Post date	Post time	
4YBY Instagram			4,442
1st post	2/5/24	18:23	
2nd POST	2/5/24	18:31	
4YBY Facebook			1,000
1st post	2/5/24	18:24	
2nd post	2/5/24	18:31	

### 2.5 Sampling and participants

This study used purposive sampling, a non-probability sampling technique, among Nigerian youth ages 14–24 who responded to the open call. Recruitment was conducted through peer youth ambassadors, social media outreach (e.g., Instagram, Facebook, WhatsApp), and the distribution of printed flyers in schools and community centers.

### 2.6 Data sources

This study utilized two data sources: a) social media engagement metrics and b) open call submission data. Specifically, two social media platforms, Instagram and Facebook, were utilized to collect engagement metrics, including post reach, engagement levels, and the number of followers for the social media accounts. Unlike Facebook or Instagram, WhatsApp does not have features to collect engagement metrics and is an end-to-end encrypted messaging platform. Additionally, the open call submission data encompassed participants’ demographic characteristics, such as age, gender, level of education, employment status, and geographical distribution.

### 2.7 Qualitative (open-ended) question

An open-ended question, “*How did you hear about the contest?”* was included to ask participants about the sources of awareness concerning the open call.

### 2.8 Ethical considerations

Ethical approval for this study was obtained as part of a larger study (IRB# 202312092), and written consent and assent were obtained from all participants (ages 14–17) who participated in our survey. Submissions were anonymized, and the data was stored securely.

### 2.9 Data analysis

For quantitative data, using R version 4.4.2, descriptive statistics were performed on social media engagement metrics and the online open call submission data. At the bivariate level of analysis, social demographic characteristics were compared between those who learned about the open call digitally and those who learned about it non-digitally. Digital platforms included responses such as Instagram, WhatsApp, Facebook, and X (formerly Twitter). Non-digital platforms encompassed responses such as flyers, banners, word of mouth, and referrals from friends and colleagues. For qualitative data (open-ended responses), Clarke and Braun's (2013) six-step data analysis process guided our analysis process (29). Initially, we thoroughly familiarized ourselves with the responses, created categories based on them, and assigned codes to one or more relevant categories. After validating the categories through team discussion, a Word Cloud was employed to illustrate the common sources of awareness related to the open call, with larger font sizes signifying a higher count of specific responses compared to smaller font sizes, followed by representative quotes. Following an independent analysis of both data sets, we merged the findings to provide a comprehensive understanding of the research.

## 3 Results

### 3.1 Digital engagement with the open call on digital platforms

**Figure 2:**
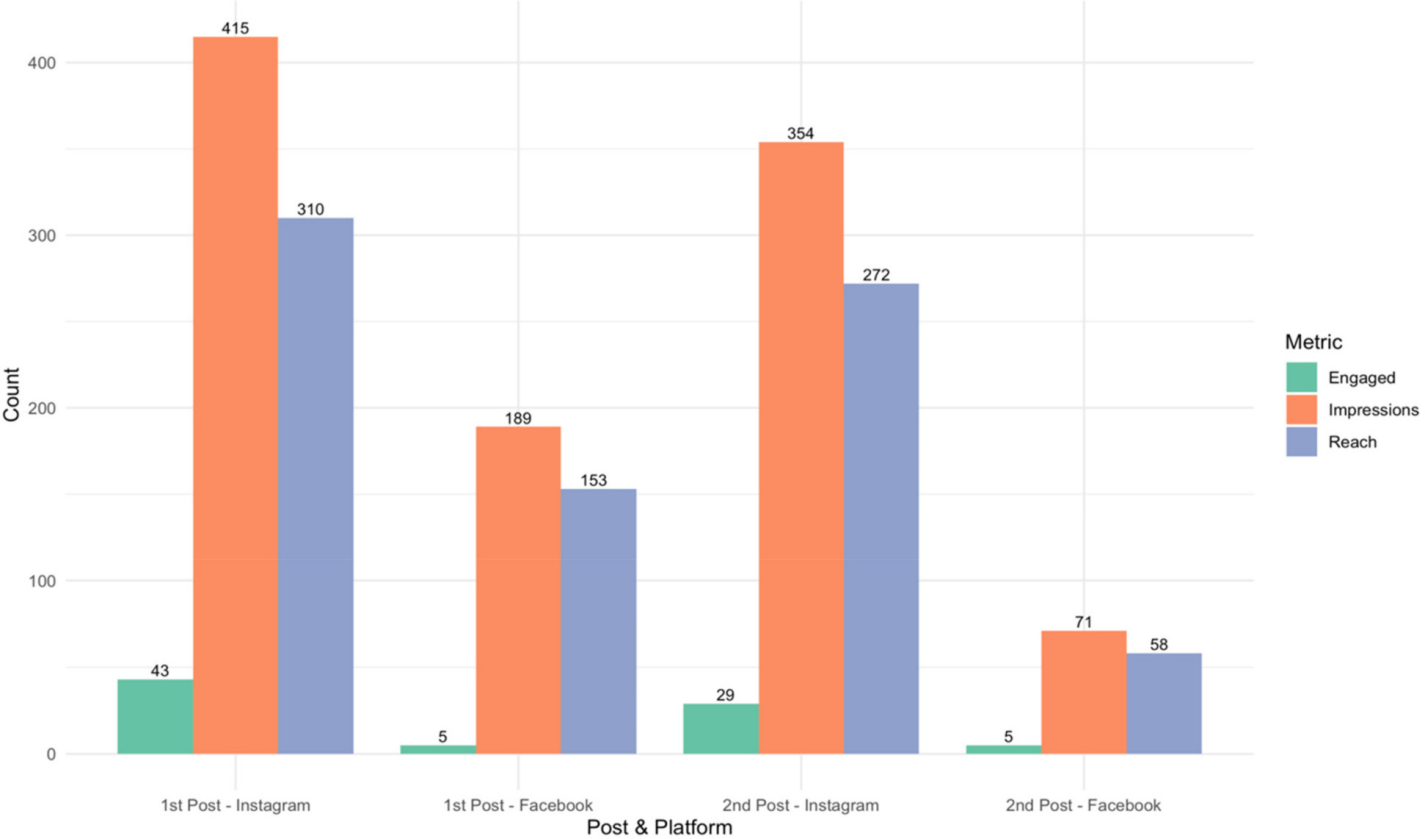
Digital engagement derived from Instagram and Facebook analytics.

Figure 2 presents a detailed overview of the digital engagement metrics from the Instagram and Facebook platforms. The first Instagram post reached approximately 310 individuals, of whom 233 (75%) were 4YBY Instagram followers. Of 43 people who interacted with the post, 35 (81%) were followers, resulting in 415 engagements. The second Instagram post reached around 272 people, with 79% of them being followers. Almost all 28 (97%) of the 29 individuals who engaged with the second Instagram post were followers, garnering about 354 impressions. Additionally, the first Facebook post reached about 153 individuals, generating five interactions and 189 impressions, while the second Facebook post reached 58 individuals, also with five interactions and 71 impressions.

### 3.2 Community participation in the open call

The community, especially AYA, responded impressively to the open call. The open call received 104 online submissions, mainly from AYA across Nigeria. The median age of the submitters was 22, ranging from 14 to 56 years old. The majority of submissions (62%) came from female participants, while 71 (68%) were students, and 45 (43%) were at the senior secondary school level. Most (57%) of submissions came from the southwest region of Nigeria, as presented in Table 2.

**Table 2:** Source of awareness and demographic characteristics of open call submissions.

Variables	Source of awareness about the open call		Overall (*N* = 104)
	Digital (*N* = 84)	Non-digital (*N* = 20)	
Age*			
Mean (SD)	22.5 (5.12)	19.8 (5.87)	21.9 (5.35)
Median [min, max]	22.0 [14.0, 56.0]	18.0 [14.0, 41.0]	22.0 [14.0, 56.0]
Gender			
Female	51 (60.7%)	13 (65.0%)	64 (61.5%)
Male	33 (39.3%)	7 (35.0%)	40 (38.5%)
Education			
Bachelors	23 (27.4%)	6 (30.0%)	29 (27.9%)
Junior secondary school	5 (6.0%)	1 (5.0%)	6 (5.8%)
Others	7 (8.3%)	1 (5.0%)	8 (7.7%)
Senior secondary school	34 (40.5%)	11 (55.0%)	45 (43.3%)
Some tertiary education	15 (17.9%)	1 (5.0%)	16 (15.4%)
Employment			
Business	1 (1.2%)	0 (0%)	1 (1.0%)
Corps member	2 (2.4%)	1 (5.0%)	3 (2.9%)
Employed	10 (11.9%)	2 (10.0%)	12 (11.5%)
Student	56 (66.7%)	15 (75.0%)	71 (68.3%)
Unemployed	14 (16.7%)	2 (10.0%)	16 (15.4%)
Volunteer	1 (1.2%)	0 (0%)	1 (1.0%)
Region			
North-central	18 (21.4%)	2 (10.0%)	20 (19.2%)
North-east	4 (4.8%)	0 (0%)	4 (3.8%)
North-west	3 (3.6%)	0 (0%)	3 (2.9%)
South-east	8 (9.5%)	2 (10.0%)	10 (9.6%)
South-south	5 (6.0%)	4 (20.0%)	9 (8.7%)
South-west	46 (54.8%)	11 (55.0%)	57 (54.8%)
Missing	0 (0%)	1 (5.0%)	1 (1.0%)

* Significant at a bivariate level of analysis at *p* = 0.001; Digital platforms included Instagram, WhatsApp, Facebook, website, X, formally Twitter, and Non-digital platforms included flyers, banners, friends, and colleagues.

### 3.3 Digital and non-digital sources of awareness regarding the open call

**Figure 3:**
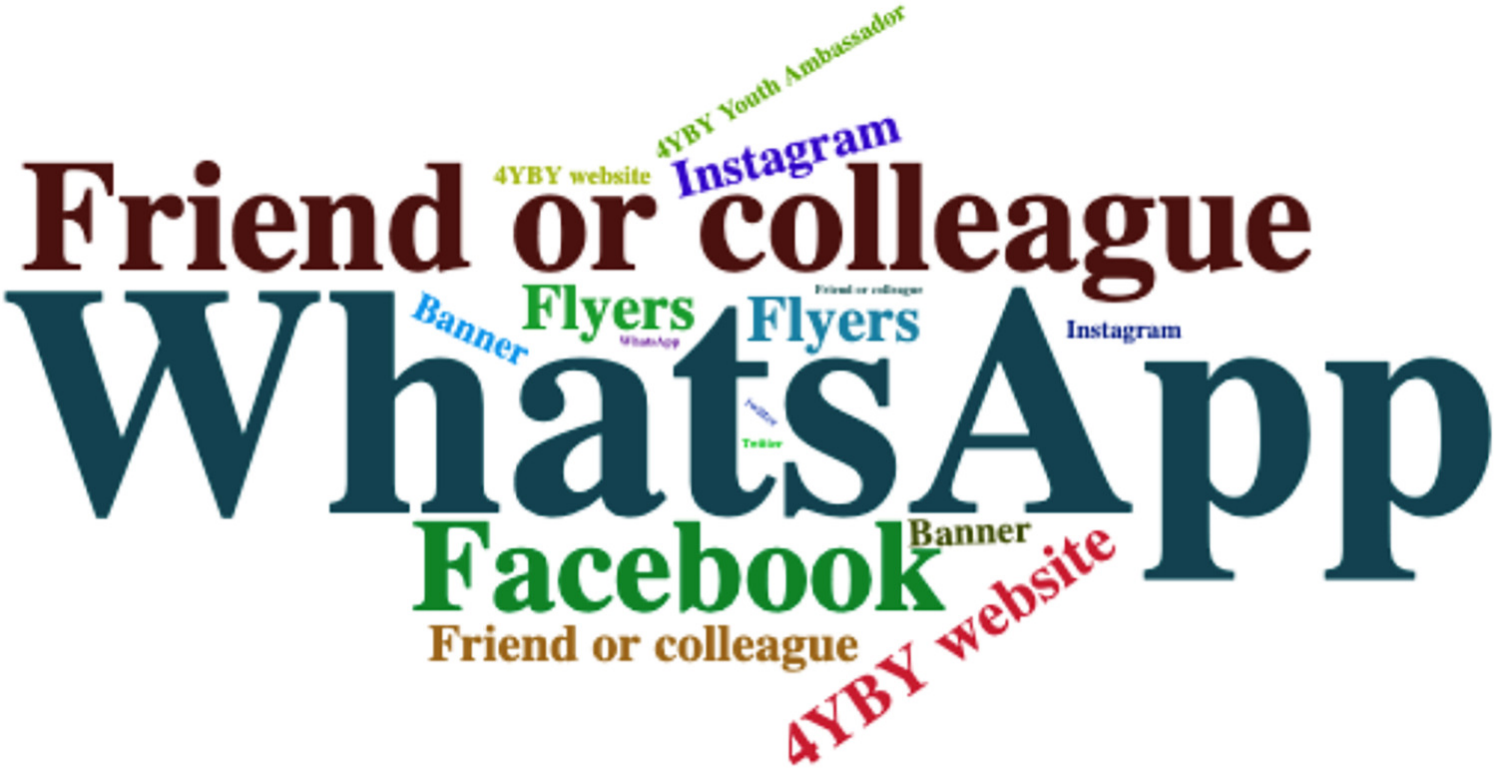
Different sources of awareness about the open call.

Individuals were asked to indicate where they heard about the open call. As shown in Table 2 and Figure 3, approximately 84 respondents (81%) reported that their sources of awareness were digital platforms, including WhatsApp, Facebook, Instagram, and the 4YBY website. Additionally, the non-digital platforms through which participants learned about the call included friends and colleagues, flyers, and banners displayed in the community by the project team. Specifically, participants stated that they had heard about the open call from multiple online and in-person sources, such as:

“I heard about the open call from WhatsApp, Facebook, and the 4YBY website,” or “I heard about it from the 4YBY Youth Ambassador, Banner, and Flyers.”

Others mentioned a combination of both online and in-person sources, stating,

“I saw it on Facebook, Instagram, WhatsApp, 4YBY website, and also from the 4YBY Youth Ambassador.”

Furthermore, the mean (SD) age of individuals who learned about the open call through digital means was 22.5 (5.12), compared to 19.8 (5.87) for those who learned about it through non-digital means. The mean difference between these two groups was significant (*p* = 0.001). Other factors, such as gender, education level, employment status, and religion, were not significant.

## 4 Discussion

Our study describes digital strategies used to engage AYA during an open call for ideas about sustaining HIV prevention programs for AYA. Our findings suggest that digital strategies can be helpful in engaging adolescents and young adults in HIV research and interventions. During the open call, digital strategies included promoting on social media platforms like Instagram and Facebook, enlisting youth ambassadors to spread the word through their networks via in-person events, social media, and WhatsApp. Additionally, participants were given the opportunity to submit their entries digitally and engage with communities on platforms such as WhatsApp groups.

Since 2019, the 4 Youth by Youth project has amassed a following across our social media pages on Instagram, Facebook, and Twitter by organizing similar crowdsourcing contests on HIV and other health themes. Many of these contests involve physical sprint events and/or boot camps where AYA learn about research and social impact while building relationships with more experienced researchers. This may partly explain why followers were more likely to interact with our content than non-followers, despite using an incentive-based promotion emphasizing the possibility of winning cash prizes.

From a study involving 1,700 social media influencers, we learn that followers may be more likely to engage with an account with a personal relationship, regardless of the account's size (30). Various factors might cause research groups or organizations to choose in-person or digital strategies to engage AYAs when they may benefit greatly from combining both. Utilizing such an approach may benefit HIV interventions by increasing community engagement and fostering a sense of community ownership (31).

Engaging with AYA through social media platforms presents an opportunity for researchers to reach a wider audience. In our study, submissions were received from every geopolitical zone in Nigeria, of which 62% were from female participants. Grove (2019) highlighted the role of social media and other digital strategies in removing barriers to research, increasing the participation of at-risk groups in research, and thereby removing inequities in research (32). In a paper comparing five case studies on the use of digital technologies in HIV interventions, authors Jones et al. conclude that digital technology could be used to ensure equitable access to HIV interventions even in low-resource settings (31). These findings indicate that digital strategies involving social media may give researchers access to a diverse group of AYA they may not be able to access ordinarily. While in-person strategies may help build relationships between research organizations and AYA, digital strategies, such as WhatsApp, Instagram, and Facebook can sustain engagement and reach marginalized populations.

Another important platform for recruitment was WhatsApp. Many of those who indicated WhatsApp as a source of awareness also reported learning about the open call from Friends or colleagues and/or 4YouthByYouth youth ambassadors. WhatsApp is a direct messaging platform that has been used by approximately 50% of Nigerian adults (33). Individuals can send information to close contacts in real time; its widespread use allows AYA to share opportunities like open calls to friends and family in their network (34). A few studies have reported the use of WhatsApp in implementation research, especially in low- and middle-income countries (LMICs). Nevertheless, the role of WhatsApp in research is under-researched (32).

The strengths of this study include utilizing digital strategies like Facebook and Instagram, which are widely used among this age group, along with participatory action research to actively engage AYA in co-creating HIV interventions for and with AYA from all six geopolitical zones in Nigeria. This approach not only amplifies their voices but also enhances the relevance and effectiveness of health interventions tailored to their needs.

One limitation was that the research did not investigate how the duration individuals spend following our pages might affect their interactions with the content. AYA who have shown interest in the organization may engage with its content differently than those who have not. Personal interactions with members of the organization may have influenced participants’ responses. This limitation restricts the generalizability of the study results and highlights the need for more research. The research setting might influence the study's results. HIV is a widely known condition in Nigeria, and this might have influenced youths’ responses to the open call. To identify other factors that might influence youth engagement, this study should be replicated in settings where this is not the case to identify other factors that might influence youth engagement.

## 5 Conclusion

As the scientific community continues to adopt participatory research approaches, there is an urgent need to explore innovative and ethical ways to engage AYA and other non-researchers in HIV prevention. Our study highlights that crowdsourcing ideas through digital platforms such as WhatsApp, Instagram, and Facebook can provide researchers with access to diverse, inclusive, and often hard-to-reach AYA populations. These platforms can foster ongoing engagement, community ownership, and the co-creation of HIV interventions tailored to the realities of young people in LMICs.

Despite the promise of digital engagement, challenges such as disparities in digital access, varying regional policies on youth-targeted content, and ethical concerns—particularly around parental consent and the age of participation—must be acknowledged. A blended approach that combines digital and in-person strategies may offer a more equitable and context-sensitive solution for sustained AYA involvement.

Looking ahead, we recommend incorporating flexible, youth-centered digital and community-based engagement models into national HIV prevention frameworks, supported by strong ethical guidelines and community partnerships. Future research should evaluate not only the effectiveness of these blended models but also their scalability, cultural appropriateness, and impact on HIV-related outcomes, in order to create a more inclusive and impactful future for HIV prevention efforts.

## Data Availability

The raw data supporting the conclusions of this article will be made available by the authors, without undue reservation.
